# Investigating Gait, Movement, and Coordination in Children with Neurodevelopmental Disorders: Is There a Role for Motor Abnormalities in Atypical Neurodevelopment?

**DOI:** 10.3390/brainsci10090601

**Published:** 2020-09-02

**Authors:** Marco Colizzi, Marco Luigi Ciceri, Gianfranco Di Gennaro, Beatrice Morari, Alessandra Inglese, Marialuisa Gandolfi, Nicola Smania, Leonardo Zoccante

**Affiliations:** 1 Section of Psychiatry, Department of Neurosciences, Biomedicine and Movement Sciences, University of Verona, 37134 Verona, Italy; 2 Department of Psychosis Studies, Institute of Psychiatry, Psychology and Neuroscience, King’s College London, London SE5 8AF, UK; 3 Child and Adolescent Neuropsychiatry Unit, Maternal-Child Integrated Care Department, Integrated University Hospital of Verona, 37126 Verona, Italy; marcoluigi.ciceri@aovr.veneto.it (M.L.C.); morari.beatrice@gmail.com (B.M.); alessandrainglese3@gmail.com (A.I.); leonardo.zoccante@aovr.veneto.it (L.Z.); 4 Neurorehabilitation Unit, Integrated University Hospital of Verona, 37134 Verona, Italy; marialuisa.gandolfi@univr.it (M.G.); nicola.smania@univr.it (N.S.); 5 Department of Pathology and Diagnostics, Integrated University Hospital of Verona, 37126 Verona, Italy; gianfradig@gmail.com; 6 Neuromotor and Cognitive Rehabilitation Research Centre (CRRNC), Department of Neurosciences, Biomedicine and Movement Sciences, University of Verona, 37134 Verona, Italy

**Keywords:** Autism Spectrum Disorder, Attention-Deficit/Hyperactivity Disorder, Tourette Disorder, transdiagnostic approach, mental health prevention

## Abstract

Motor abnormalities have been suggested to play a role in most neuropsychiatric disorders, as a potential generic neurodevelopmental vulnerability. However, they still represent a neglected area, with a paucity of empirical studies, especially in pediatric populations. This case-control study aimed to comprehensively assess motor functioning in children with atypical neurodevelopment and investigate whether any socio-demographic or clinical characteristics would concur with motor difficulties to distinguish children with neurodevelopmental disorders (NDD) from healthy controls. Socio-demographic (age and gender) and clinical (intelligence quotient, gait, movement, and coordination) data were collected on 114 children aged 5–15 (83 with NDD, 31 healthy controls). Male children were at significantly higher risk for NDD (OR: 13.023, *p* < 0.001). Furthermore, there was a statistically significant interaction between the total intelligence quotient and overall coordination such that increasing levels of total intelligence quotient appeared to protect against the likelihood of being diagnosed with an NDD, but only in the context of a preserved coordination (OR: 0.964, *p* = 0.038). Collectively, results may have important public health implications, as they point towards the development of new approaches to establish an early prognosis in neurodevelopment, including assessing motor difficulties and mitigating their impact on children’s quality of life.

## 1. Introduction

Autism Spectrum Disorder (ASD), Attention-Deficit/Hyperactivity Disorder (ADHD), and Tourette Disorder (TD) have been historically classified as distinct disorders, although with the commonality of childhood onset [1]. However, their separation into categorical diagnoses has been questioned [2], due to the evidence of substantial comorbidity [3,4,5] and overlap in terms of neuropsychological and psychopathological manifestations [4,6,7,8] as well as neurobiological and genetic underpinnings [9,10,11,12]. Clinical research evidence has led to the gathering of such conditions into the single overarching category of neurodevelopmental disorders (NDD), along with other intellectual functioning disorders such as communication and learning disabilities [13], also putting them in the neurodevelopmental continuum with disorders that typically emerge in late adolescence and early adulthood such as affective and non-affective psychoses [1].

Such evidence-based change in nosology urges that the diagnosis and treatment of NDD should involve the assessment and recognition not only of the core features of each single disorder, but also of symptoms and difficulties frequently associated or reported across the conditions [2]. For instance, an impairment in socialization is a core feature of ASD [13] but also frequently reported in ADHD [4,7,14] and TD [15]. Similarly, hyperactivity and impairment in control are core features of ADHD [16], but associated symptoms of ASD [16] and TD [5]. Further, anxiety [14,17], depressive symptoms [14,17,18], obsessive-compulsive behaviors [5,16], and sleep disturbances [14,19,20,21] are trans-diagnostic features associated with all three neurodevelopmental conditions. Overall, it seems to be a necessary step to reorient the clinical research of NDD in an attempt to unravel the trans-diagnostic nature of their neuropathology.

Motor abnormalities have been suggested not to be disorder-specific but rather a core phenotype dimension that cuts across neuropsychiatric disorders [22,23]. In fact, they are included among the diagnostic criteria of many conditions, and NDD are no exception, as stereotypic movements are diagnostic of ASD, hyperactivity of ADHD, and tics of TD [13]. Despite research evidence supporting a role of motor abnormalities in the etiology [24,25], nosology [26,27], pathophysiology [28], and management [29,30] of neuropsychiatric disorders, they still represent a neglected area in both clinical practice and research [31,32], with most research evaluating motor functioning mainly focusing on psychosis [33] and a paucity of studies comparing motor abnormalities across different conditions [22].

In light of the lack of empirical studies systematically examining motor function across different neuropsychiatric conditions, especially NDD, the present study attempted to fill this gap by conducting a comprehensive assessment of motor abilities across different domains such as gait, movement, and coordination, in a group of young individuals with NDD as compared to a group of healthy controls. We hypothesized that NDD individuals would present with poorer motor functioning than healthy controls in terms of (i) less proficient spatiotemporal gait organization, indexed by an increased step time as the most investigated [34] and reliable [35] measure of less efficient gait patterning [36]; (ii) less proficient motor skills, indexed by a lower total score at the Movement Assessment Battery for Children, 2nd edition (M-ABC-2) [37]; and (iii) less proficient coordination, indexed by a lower total score at the Developmental Coordination Disorder Questionnaire, as revised in 2007 (DCDQ’07) [38]. Furthermore, we conducted exploratory analyses in order to investigate whether any socio-demographic or clinical characteristics would concur with anomalies in motor functioning to distinguish NDD individuals from healthy controls, especially male gender and dysfunctional neurocognition which are known to play a key role in atypical development [13].

## 2. Materials and Methods

### 2.1. Participants

Participants were recruited as part of a larger case-control study, carried out at the Veneto Autism Spectrum Disorder Regional Centre of the Integrated University Hospital of Verona, Italy. Children aged 5–15 years (the age range for which the movement and coordination instruments have been validated) were screened for a neurodevelopmental disorder (NDD) and included if meeting the diagnostic criteria for Autism Spectrum Disorder (ASD), Attention-Deficit/Hyperactivity Disorder (ADHD), or Tourette Disorder (TD), according to the Diagnostic and Statistical Manual of Mental Disorders, 5th edition, (DSM-5) diagnostic criteria. Exclusion criteria were (i) formal NDD comorbidity, i.e., fulfilling DSM-5 diagnostic criteria for two or more different NDD (e.g., fulfilling DSM-5 diagnostic criteria for both ADHD and TD); (ii) formal neuropsychiatric comorbidity, i.e., fulfilling DSM-5 diagnostic criteria for other mental disorders such as schizophrenia spectrum and other psychotic disorders, depressive disorders, anxiety disorders, and obsessive-compulsive and related disorders (e.g., fulfilling DSM-5 diagnostic criteria for both ASD and Obsessive Compulsive Disorder); (iii) any significant medical illness, especially a neurological (e.g., cerebral palsy, epilepsy, or otherwise-classified motor handicap) or orthopedic (e.g., fracture, severe injury) condition; (iv) evidence of a genetic syndrome (e.g., chromosomal abnormalities); (v) severity of the condition such as to preclude the ability to complete the expected assessments. 

Healthy control subjects were recruited thanks to the collaboration with a number of primary and secondary schools of the city of Verona as well as the Hospital Pediatric Unit. Those willing to take part in the study were included provided that they were in good overall health and did not satisfy the inclusion criteria for any NDD. As for NDD children, potential control subjects were excluded if they met DSM-5 diagnostic criteria for any other neuropsychiatric disorder or had any significant medical illness or genetic syndrome.

### 2.2. General Assessment

Sociodemographic data, including age and gender, were collected on all NDD children and control subjects. All subjects underwent an extensive clinical evaluation with experienced physicians, based on analysis of any medical documentation, in-depth physical examination, anamnestic interviews with parents/guardians and children, and assessment of the child’s abilities through scales. A cognitive evaluation was performed with the Wechsler Intelligence Scale for Children, 4th edition (WISC-IV).

### 2.3. Motor Functioning

#### 2.3.1. Spatiotemporal Gait Organization

Children’s gait was evaluated using the GAITRite electronic walkway system (GAITRite Platinum; CIR Systems, Franklin, NJ, USA), consisting of a walkway 7.92 m in length and sampling at a frequency of 120 Hz, with integrated pressure sensors, as done in previous studies in similar populations of children with NDD [34]. The reliability of such tool to measure gait parameters in pediatric populations has been proved [39]. As per protocol, participants were asked to perform six walking trials at their normal pace. By taking into account participants’ height, weight, and bilateral femoral-tibial length, the following spatiotemporal measures of gait were estimated from the system: (i) velocity (cm/s); (ii) cadence (step/min); (iii) step time (s; time from initial contact of one foot to initial contact of the other foot); (iv) step length (cm; distance from the point of initial contact of one foot to the point of initial contact of the other foot); (v) stance phase (% of the total gait cycle; supporting phase of the gait cycle, from the first to the last contact of two consecutive supports of the same foot); (vi) swing phase (% of the total gait cycle; phase of the gait cycle without support, from the last contact of the first support to the first contact of the following support of the same foot); (vii) single support phase (% of the total gait cycle spent supported by only one foot); (viii) double support phase (% of the total gait cycle spent supported by both feet). For further data analysis, gait parameters were then averaged over the six walking trials and, when appropriate, over the two limbs. 

When assessing gait outcome, step time was considered of particular interest, as it is one of the most investigated measures in neurodevelopment [34] and evidence suggests it to be a specific indicator of gait patterning [36], while other measures may be more useful when assessing functional performance (e.g., velocity) [40] or equilibrium (e.g., support phases) [41]. Moreover, along with cadence, step time has been recently suggested to be the most robust parameter in terms of reproducibility, showing good intraclass correlation coefficient, and yielding a false positive in terms of gait deficits less likely [35]. By contrast, other parameters (e.g., velocity, step length) may change depending on the condition and/or intention of the subjects participating in the gait measurement, significantly increasing the chances of yielding a false-positive result [35].

#### 2.3.2. Movement

Children’s motor function was assessed using the Movement Assessment Battery for Children, 2nd edition (M-ABC-2), an individually administered standardized measure of motor function for children aged 3–16 years [37]. The M-ABC-2 is one of the most widely used instruments to identify and describe children’s motor difficulties, with established reliability and validity, and it has been already used to assess motor skills in children with NDD [34]. The tool offers three age-related item sets, each consisting of eight tasks measuring: (i) manual dexterity (three tasks); (ii) ball skills (two tasks); and (iii) balance (three tasks). Item scores can be combined to form an overall score as a general marker of motor skills, with a normative mean of 10 and standard deviation of 3. The higher is the score, the greater are the child’s motor skills. 

#### 2.3.3. Coordination

Children’s coordination was assessed using the Developmental Coordination Disorder Questionnaire, as revised in 2007 (DCDQ’07), a parent questionnaire designed as a screening tool for coordination difficulties in children aged 5–15 years [38]. Its reliability and validity have been well established [42], also in Italian populations of children [43]. The tool consists of 15 items, which group into three distinct factors: (i) control during movement; (ii) fine motor and handwriting; and (iii) general coordination. Each item is scored on a five-point Likert scale, leading to a total score ranging from 15 to 75 points. The higher the score, the greater is the child’s coordination.

### 2.4. Data Analysis

All collected variables were summarized by mean, standard deviation, median, interquartile range, minimum and maximum values. Categorical variables were expressed as percentages.

Shapiro–Wilk tests were performed to assess normal distribution of continuous variables. A binomial multiple logistic regression analysis was used to identify the predictors of the case-control status (gender, Intelligence Quotient (IQ), step time, DCDQ’07 total score, and M-ABC-2 total score). The estimating accuracy of the candidate models was measured by C-statistics. Hosmer–Lemeshow test was used to assess the model goodness-of-fit. Sensitivity analysis was run to confirm model estimates after removing highly influential patients identified by residuals examination. Assumption of linearity of independent variables and log-odds was assessed by Box-Tidwell transformation. Continuous variables were centered on their means to reduce possible multicollinearity. The variable full-scale Intelligence Quotient (FSIQ), i.e., the WISC-IV composite score that represents general intellectual ability, was introduced in the model by using a 10-point metrics to obtain more clinically interpretable results. Step time variable was introduced after reciprocal transformation to account for the pronounced skewness. The null hypothesis of no prediction was rejected when odds ratios were significantly different than one. A 5% statistical significance was set. Missing data were handled by listwise deletion. Statistical analysis was carried out by statistical package STATA (version 14).

### 2.5. Ethics

Protocols and procedures were approved by the research ethics committee at the Integrated University Hospital of Verona (CESC 2242 and CESC 2243). After complete description of the study, informed written consent was obtained from parents or guardians of all cases and control subjects included. By signing the document, they consented to the publication of data originating from the study.

## 3. Results

### 3.1. Socio-Demographic and Clinical Characteristics

A total of 114 children participated in the study, 83 children with a neurodevelopmental disorder (NDD) and 31 healthy controls. The median age of cases and controls was 10.07 (IQR: 8.48–11.72; range: 5.58–14.88) and 11.30 (IQR: 9.14–13.28; range: 7.23–15.23) years, respectively. Male children represented 38.71% of the control group and 85.54% of the case group. 

As expected, healthy controls showed an intelligence quotient in the average range, movement skill total score substantially overlapping with the normative mean of 10 and standard deviation of 3, and coordination abilities safely above performance warranting the investigation of developmental coordination difficulties. Instead, children with NDD showed an intelligence quotient in the average range, but closer to the lower limit, movement skills which were lower than previously published normative data, and coordination abilities suggestive of potential developmental coordination difficulties. Table 1 reports descriptive statistics of all collected clinical data.

### 3.2. Predictors of Case-Control Status

**Figure 1:**
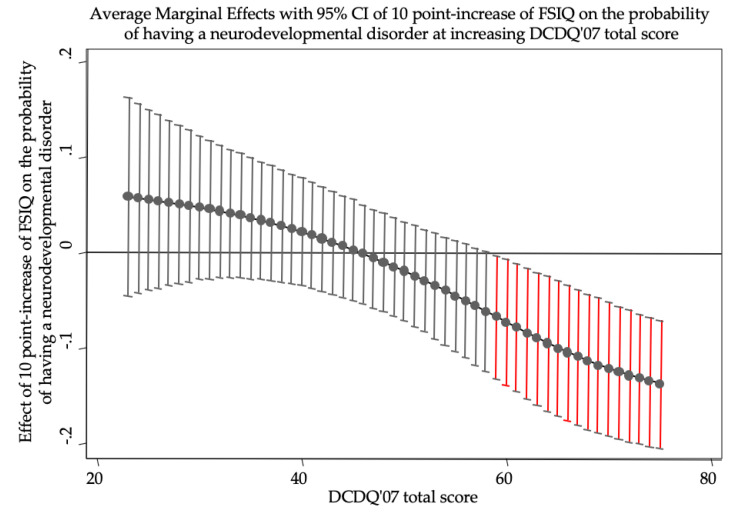
The figure shows the effects of ten-point increase in FSIQ on the probability of having a neurodevelopmental disorder when DCDQ’07 total score is held constant at different values. FSIQ has a statistically significant role (95% CIs do not reach zero probability, red bars) only at high levels of DCDQ’07 total score. At maximum levels of DCDQ’07, a ten-point increase in FSIQ lowers the probability of having a neurodevelopmental disorder of more than 10%. In odds ratio metrics, a ten-point increase in FSIQ constantly lowers the odds of having a neurodevelopmental disorder of 3.6% ((1–0.964) × 100) when DCDQ’07 increases of 1 point. FSIQ, Full Scale Intelligence Quotient; DCDQ’07, Developmental Coordination Disorder Questionnaire, as revised in 2007.

Logistic analysis (*n* = 100) highlighted male gender (OR: 13.023, *p* < 0.001) as a statistically significant predictor of having an NDD. Furthermore, the model indicated a statistically significant interaction between total IQ and DCDQ’07 total score (OR: 0.964, *p* = 0.038; Table 2). In particular, the interaction revealed a statistically significant protective role of total IQ but only at higher levels of DCDQ’07 total score (Figure 1). 

Four patients were shown to be highly influential (standardized residuals lower than −2). When removed from the analysis, model estimations were unchanged. The model selection was based on Akaike Information Criterion. The model showed a satisfying (*p* = 0.181) goodness-of-fit and a 0.862 C-statistic.

## 4. Discussion

To the best of our knowledge, this is the first case-control study to examine whether a less proficient motor function, as evaluated at the clinical, instrumental, and anamnestic level, could be a trans-diagnostic component of the overarching category of neurodevelopmental disorders (NDD). Results suggest that coordination proficiency and cognitive performance interact on the likelihood of having an NDD. In particular, increasing levels of total intelligence quotient appear to protect against the likelihood of being diagnosed with an NDD, but only in the context of a preserved coordination. In other words, the child’s total intelligence quotient is not per se sufficient to protect against an atypical development, without taking into account their coordination abilities.

The finding that the DCDQ’07 was the only instrument able to differentiate NDD children and controls in their motor functioning is not surprising. As a parent questionnaire investigating child’s difficulties in day-to-day functioning, it has the double advantage to collect information from the most reliable respondents to report developmental problems of the child and in the context of an extensive observation of the impact of potential coordination difficulties on daily living tasks [38]. Results extend previous literature regarding the usefulness of such self-report questionnaires as integration of motor performance batteries and instruments in order to help the healthcare professional to paint a complete picture of the child motor functioning [44]. 

The current classification of neuropsychiatric disorders observes the presence of one or more motor-related features in most neurodevelopmental conditions with childhood to early adulthood onset [13]. However, the lack of a scientific consensus about the role of non-normative motor functioning during brain maturation [45] and when to consider it to be pathological [46] as well as the fragmentation of research studies mainly focusing on predefined motor domains [22] has dramatically slowed down our understanding of such phenomena. In the absence of localized lesions or identified abnormalities of the brain, motor difficulties are not considered of pathological relevance in early childhood [47]. However, their persistence in late childhood may reflect a maturational delay affecting the child’s sensory integration, motor coordination, and sequencing of complex motor acts [48,49]. While it is sufficiently established that such atypical maturation may continue throughout childhood and adolescence, increasing the risk of poor psychosocial adjustment [50] because of biological (e.g., delayed or problematic brain development affecting language and sphincter control [51,52]) and psychological factors (e.g., rejection, social withdrawal, feelings of failure, low self-esteem [53,54]), we currently struggle in clinical practice to distinguish pathognomonic from non-specific or benign motor manifestations [55]. Furthermore, such motor impairments have often been suggested to specifically lie in the evolutionary trajectory to psychosis, being already present in the early phase of the disorder as well as in relatives of patients and in people at risk to develop psychosis, and to correlate with the symptom severity [56]. However, other research evidence questioned whether motor impairment would rather represent a generic neurodevelopmental vulnerability, which is not related to a single neurodevelopmental condition [56]. For instance, poorer early motor coordination has been linked with a higher risk of major depressive disorder and generalized anxiety disorder later in life [57]. Noteworthy, childhood neurodevelopmental difficulties on their own have been associated with a higher risk to present in adolescence with mental health difficulties other than psychosis, such as depression and anxiety [58]. Nevertheless, despite evidence is still limited, literature indicates that coordination difficulties may sustain negative biobehavioral trajectories [53,54], highlighting the importance of developing proficient motor skills in order for the child to be and remain in good physical and mental health throughout their life [59].

The neurodevelopmental continuum points in the direction of redefining and rendering more flexible the diagnostic and therapeutic approach to such conditions in order to identify new and trans-diagnostic biomarkers for patient stratification. There is a strong evidence for pleiotropy between ASD, ADHD, and TS, where also cognitive impairments would represent an additional outcome of the same risk for atypical neurodevelopment [60]. The results of the present study extend such findings, suggesting a crucial role for motor impairments, especially inefficient coordination, in the atypical development leading to NDD, even at increasing levels of cognitive performance. 

The present findings have at least three important clinical implications. First, rather than being etiologically discrete entities, ASD, ADHD, and TS are better conceptualized as lying on an etiological neurodevelopment continuum [58], where even the manifestation of motor symptoms characteristic of each condition may reflect the severity, timing, and predominant pattern of atypical brain development and resulting biobehavioral expressions. Stereotypic movements, hyperactivity, and tics are currently defined based on a limited comprehension of their nature. Further studies are required to better characterize the phenotype and neurobiology of such phenomena in the wider context of neurodevelopmental motor alterations. Second, most interventions in NDD focus on psychological and behavioral symptoms, learning abilities, and language fluency. Evidence suggests little room for interventions dedicated to improving motor skills, even in those neurodevelopmental conditions where poor motor proficiency is the main issue (e.g., Developmental Coordination Disorder) [61]. Future studies need to longitudinally evaluate the impact of early intervention programs to mitigate motor difficulties in neurodevelopment, also in terms of other psychosocial and neurocognitive parameters of severity and outcome. Third, approaches may be fruitful across diagnostic boundaries in neurodevelopment and future studies will estimate their effectiveness in light of the neurodevelopmental gradient hypothesis that the higher the psychopathological, cognitive, genetic, and sensorimotor severity itself, the greater the neurodevelopmental impairment [60].

The empirical results reported herein should be considered in the light of some strengths and limitations. The main strengths of the study are the adoption of strict inclusion criteria in terms of diagnostic groups, and the detailed assessment of motor abilities. Such aspects of the study design allow excluding that the evidence of motor difficulties in NDD would be attributable to other medical or psychiatric comorbidities or a bias due to a partial assessment. Consistently, we also adopted a cautious approach when selecting parameters indicative of motor dysfunction, especially in terms of gait patterning. The main limitations of the study are that its sample was too limited to fully investigate the predictive value of motor skill impairments in each NDD as well as how motor function would change as a function of age, also reporting a static representation of the phenomenon in neurodevelopment. Future longitudinal studies in larger populations may be able to track the atypical trajectory of neurodevelopment as a function of less proficient motor abilities as well as evaluate the stability of such phenomena from childhood throughout adolescence [47]. Moreover, the case-control imbalance in terms of sample size and gender distribution may have led to a reduced precision of the estimates of the investigated effects, warranting further studies in larger populations in order to investigate the association between coordination and intellectual ability as a function of gender.

## 5. Conclusions

In summary, while awaiting replication, the study results suggest that poor coordination may be a marker of risk for NDD, even in the presence of substantially intact cognitive function, thus providing novel insight into the neurobiology of atypical neurodevelopmental trajectories. Evidence for a role of coordination difficulties in atypical neurodevelopment is also in line with the hypothesis of soft neurological signs in neuropsychiatric disorders [62]. Collectively, this study may have important public health implications, as it points towards the development of new approaches to establish an early prognosis in neurodevelopment, including the assessment of motor difficulties and the mitigation of their impact on quality of life.

## Figures and Tables

**Table 1:** Clinical characteristics of the study sample.

	NDD Individuals				Healthy Controls			
	n	M (SD)	Median (IQR)	Range	n	M (SD)	Median (IQR)	Range
**WISC IV**								
Verbal Comprehension	82	98.93 (15.41)	97 (90–110)	56–142	30	102.87 (14.51)	103 (90–110)	80–140
Perceptual Reasoning	82	103.73 (17.46)	105 (93–115)	54–141	30	110.23 (10.03)	111 (104–115)	91–132
Working Memory	82	89.13 (13.93)	88 (79–100)	52–127	30	99.60 (12.50)	103 (88–109)	79–121
Processing Speed	81	89.54 (18.14)	88 (76–100)	47–129	30	102.27 (17.39)	103 (91–115)	56–132
full-scale Intelligence Quotient	81	95 (17.10)	95 (86–107)	45–141	30	105.83 (12.45)	104 (97–115)	79–132
**Spatiotemporal gait organization**								
Velocity (cm/s)	79	102.64 (17.61)	102.4 (89.2–114.7)	63.2–143.3	31	108.76 (19.16)	112.1 (89.7–121.5)	74.2–149.4
Cadence (step/min)	79	110.94 (13.69)	110.2 (101.3–119)	78.5–145.9	31	112.35 (10.51)	112.2 (104.5–117.2)	91–138.2
Step time (s) *	79	0.55 (0.07)	0.54 (0.5–0.59)	0.41–0.76	31	0.55 (0.11)	0.53 (0.51–0.58)	0.43–1.09
Step length (cm)	79	55.53 (7.09)	54.53 (50.27–59.52)	43.11–77.67	31	58.13 (8.91)	59.36 (52.1–65.07)	41.25–73.91
Stance phase (%)	79	61(1.51)	61.15 (60–62.05)	57.95–64.15	31	60.45 (1.26)	60.2 (59.3–61.65)	58–63.1
Swing phase (%)	79	38.99 (1.51)	38.85 (38–40.05)	35.85–42.05	31	39.56 (1.28)	39.8 (38.4–40.7)	36.85–42
Single support phase (%) *	79	39.36 (4.89)	38.85 (38–40.1)	26.25–78.3	31	39.55 (1.28)	39.8 (38.4–40.65)	36.75–42.05
Double support phase (%)	79	21.58 (2.99)	21.5 (19.5–23.8)	14.7–27.85	31	20.50 (2.37)	20.2 (18.45–22.8)	15.55–24.25
**M-ABC-2**								
Manual dexterity *	82	8.39 (4.48)	8 (5–11)	1–19	31	10.23 (3.44)	11 (8–13)	4–16
Ball skills	82	8.38 (3.14)	8 (7–10)	2–19	31	10.13 (3.29)	11 (8–12)	1–16
Balance	82	9.95 (4.88)	9 (6–14)	1–19	31	11.29 (3.23)	11 (10–14)	3–16
Total score	82	8.78 (4.54)	8.5 (5–12)	1–19	31	10.74 (3.71)	11 (8–14)	3–16
**DCDQ’07**								
Control during movement	77	20.27 (5.39)	19 (16–25)	9–30	29	23.52 (5.51)	24 (21–28)	10–30
Fine motor and handwriting	77	12.82 (4.13)	13 (9–16)	4–20	29	16.55 (3.25)	17 (14–20)	10–20
General coordination	77	14.79 (4.18)	14 (12–17)	5–25	29	18.97 (4.12)	20 (15–22)	11–25
Total score	77	47.88 (11.17)	48 (41–54)	23–74	29	59.03 (12.14)	62 (52–70)	32–75

NDD, Neurodevelopmental Disorders; M, mean; S.D., standard deviation; IQR, interquartile range; * Skewed data as for Shapiro–Wilk test for normal data; WISC-IV, Wechsler intelligence scale for children-fourth edition; M-ABC-2, Movement Assessment Battery for Children, 2nd edition; DCDQ’07; Developmental Coordination Disorder Questionnaire 2007.

**Table 2:** Predictors of case-control status.

	*p*-Value	OR	95% CI
Gender	<0.001	13.023	3.693–47.080
Step time	0.661	1.690	0.161–17.705
M-ABC-2 total score	0.361	0.929	0.794–1.087
DCDQ’07 total score	0.054	0.945	0.891–1.001
full-scale Intelligence Quotient	0.368	0.823	0.539–1.257
DCDQ’07 total score * full-scale Intelligence Quotient	0.038	0.964	0.931–0.998

OR, odds ratio; CI, confidence interval; M-ABC-2, Movement Assessment Battery for Children, 2nd edition; DCDQ’07, Developmental Coordination Disorder Questionnaire 2007; *, interaction.
